# Isokinetic Performance of Shoulder External and Internal Rotators of Professional Volleyball Athletes by Different Positions

**DOI:** 10.1038/s41598-020-65630-9

**Published:** 2020-05-26

**Authors:** Do-Kyung Kim, Geon Park, Liang-Tseng Kuo, Won-Hah Park

**Affiliations:** 1 Department of Sports Medicine Center, Samsung Medical Center, Sungkyunkwan University School of Medicine, Seoul, Korea; 2 Sports Medicine Center, Department of Orthopaedic Surgery, Chang Gung Memorial Hospital, Chiayi, Taiwan; 3 School of Medicine, College of Medicine, Chang Gung University, Taoyuan City, Taiwan

**Keywords:** Musculoskeletal system, Muscle, Orthopaedics, Medical research

## Abstract

This study aimed to exam the isokinetic shoulder rotator strength of professional volleyball athletes, by playing positions. This cross-sectional study included a total of 49 healthy male professional volleyball players. We measured the isokinetic strength of the external rotator (ER) and internal rotator (IR) muscles and compared the dominant and non-dominant shoulders at angular speeds of 60°/s and 180°/s. In ER, all positions of players had similar strength between the dominant shoulder and non-dominant shoulders. Conversely, all playing positions except libero had stronger strength in dominant shoulder than that in the non-dominant shoulder. The ER/IR ratio in the dominant shoulder was significantly lower only for the attacker (outside hitter and opposite) at 60°/s and 180°/s (P < 0.0001; P = 0.0028 respectively) and blocker at 60°/s (P = 0.0273) when compared with non-dominant shoulder. Furthermore, the attacker had a lower ER/IR ratio in the dominant shoulder than setter and libero at 60°/s and 180°/s. For elite volleyball players without injury, the dominant shoulder had a higher strength of internal rotation, causing the relative muscle imbalance than the non-dominant shoulder, especially for the attacker and blocker positions. Training program should be individualized for each playing position to improve the imbalanced shoulder.

## Introduction

Volleyball is considered to be one of the fastest-paced sports requiring highly developed qualities of strength, power, agility, and speed. To increase performance, volleyball players need to jump higher and spike balls stronger and faster than any other sports. The shoulder joint receives the extreme load during repetitive volleyball movements; consequently, many injuries affect this joint in volleyball players^1^. Eventually, the excessive external rotation associated with primary volleyball techniques is known to overload the surrounding muscles and ligaments, causing injury^2,3^. Continuous overhead movements induce centrifugal force from forceful eccentric contractions of rotator cuff muscles on the glenohumeral joint, leading to micro-damage to the muscles and limited joint movements^4–6^. Previous studies suggested that the differences in internal rotator (IR) and external rotator (ER) muscle strength ratios appear to be related to injury in almost all sports that involve overhead throwing activities, such as baseball, tennis, and volleyball^7–10^.

The previous study had evaluated the existence of strength asymmetry of the ER and IR muscles in professional volleyball players^11^. There are four different positions of players in the volleyball game: attackers (outside hitter and opposite), main blocking and attacking middle blocker, overhand passing for toss setter, and defending libero^12^. Although there are considerable differences in physical characteristics among different positions of volleyball players, comparative studies of the asymmetric shoulder features of each position are lacking. Therefore, we designed this study to exam the shoulder rotator muscle strength in four positions of volleyball players, including attackers, blocker, setter, and libero.

This study aimed to establish the concentric strength profiles of the IR and ER muscles in healthy male professional volleyball players by positions and to evaluate the ER/IR strength ratios of the different playing positions. We hypothesized that the different positions would have different IR/ER muscle strength ratios in the shoulder joint due to the differences in specific tasks and movements such as toss, spike, serve, block, and dig.

## Results

From January 2014 to December 2018, we totally included 49 healthy male players of the professional volleyball team in this study, among whom, 19 attackers, ten blockers, ten setters, and ten liberos. The average age was 23.5 ± 3.3 years. The average height was 190 ± 8.3 cm. Thirty-five players were right-handed, and the other 14 players were left-handed. The characteristics of the study participants were shown in Table 1.

**Table 1:** Participants characteristics.

Characteristic	Participant data (n = 49)^a^
Age, years	23.5 ± 3.3
Height, cm	190.0 ± 8.3
Weight, kg	83.1 ± 8.7
BMI, kg/m^2^	22.9 ± 1.4
**Player position, n (%)**	
Attacker	19 (38.7)
Blocker	10 (20.4)
Setter	10 (20.4)
Libero	10 (20.4)
**Hand dominance, n (%)**	
Left	14 (28.6)
Right	35 (71.4)

^a^Values are expressed as mean ± standard deviation or number (percentage).

Isokinetic tests were performed to evaluate the differences in muscle strength between the dominant and non-dominant shoulders in each player. In terms of ER, the attackers had a relatively weak strength on the dominant shoulder versus non-dominant shoulder, though not reaching the significant difference (60°/s, P = 0.2413; 180°/s, P = 0.1564). Moreover, the other three positions of players had similar ER muscle strength on the dominant side versus as non-dominant shoulder, regardless of which angular velocities (all P > 0.05, Table 2).

**Table 2:** External and Internal Rotator Strength of Dominant and Non-dominant Shoulders.

	External rotation (N-m/kg)^a^		P value	Internal rotation (N-m/kg)^a^		P value
	Dominant	Non-dominant		Dominant	Non-dominant	
**60°/sec**						
Attacker	0.32 (0.30–0.39)	0.35 (0.28–0.41)	0.2413	0.76 (0.62–0.81)	0.69 (0.51–0.75)	<0.0001*
Blocker	0.33 (0.29–0.37)	0.33 (0.31–0.38)	0.6250	0.64 (0.56–0.70)	0.61 (0.53–0.67)	0.0273*
Setter	0.32 (0.30–0.33)	0.32 (0.28–0.34)	0.2754	0.56 (0.51–0.62)	0.55 (0.47–0.57)	0.0195*
Libero	0.36 (0.32–0.39)	0.36 (0.31–0.38)	1.0000	0.58 (0.56–0.65)	0.65 (0.53–0.66)	0.8457
**180°/sec**						
Attacker	0.26 (0.25– 0.31)	0.29 (0.24–0.31)	0.1564	0.62 (0.55–0.70)	0.59 (0.49–0.63)	0.0002*
Blocker	0.29 (0.24–0.31)	0.28 (0.25–0.31)	0.8203	0.56 (0.48–0.62)	0.53 (0.49–0.55)	0.1309
Setter	0.26 (0.25–0.28)	0.27 (0.23–0.29)	0.1641	0.48 (0.44–0.53)	0.46 (0.42–0.52)	0.0391*
Libero	0.31 (0.26–0.34)	0.31 (0.27–0.34)	0.7695	0.56 (0.52–0.62)	0.56 (0.52–0.60)	0.2500

^a^Values are presented as median (interquartile range).

^*^P < 0.05.

In terms of IR, the muscle strength in the dominant shoulder was significantly higher than that in the non-dominant shoulder for all positions of players at an angular velocity of 60°/s, except libero (attacker, P < 0.0001; blocker, P = 0.0273; setter, P = 0.0195, Table 2). At 180°/s, the significant higher IR strength of the dominant side was also observed in attacker and setter (P = 0.0002, P = 0.0391, respectively, Table 2). The attacker had the highest average IR power among all positions. The results of the isokinetic IR and ER strength tests were shown in Table 2.

We further compared the ER/IR ratio between the dominant and non-dominant shoulders. At 60°/s and 180°/s, the attacker had a significantly lower ER/IR ratio of the dominant shoulder than that of the non-dominant shoulder (P < 0.0001, P = 0.0028, respectively, Table 3). The blocker had a significantly lower ER/IR ratio of the dominant shoulder than that of the non-dominant shoulder, at 60°/s, but not at 180°/s (P = 0.0273, P = 0.0645, respectively, Table 3).

**Table 3:** External/Internal Rotator Strength Ratios of the Dominant and Non-dominant Shoulder.

	ER/IR ratio (%)^a^		P value
	Dominant	Non-dominant	
**60°/sec**			
Attacker	48.1 (41.2–51.2)	55.5 (49.3–57.6)	<0.0001*
Blocker	51.2 (48.0–56.5)	58.3 (54.7–59.9)	0.0273*
Setter	57.2 (53.1–62.1)	59.3 (52.1–65.5)	0.1934
Libero	55.9 (50.0–65.6)	57.5 (53.1–62.9)	0.8457
**180°/sec**			
Attacker	43.8 (41.4–47.8)	49.4 (44.7–53.6)	0.0028*
Blocker	47.0 (45.8–57.4)	53.2 (48.2–59.4)	0.0645
Setter	55.3 (51.4–57.3)	57.4 (51.1–58.9)	0.3750
Libero	53.1 (49.2–57.8)	54.4 (52.3–60.3)	0.1602

ER, external rotator; IR, internal rotator.

^a^Values are presented as median (interquartile range).

*P < 0.05

**Figure 1:**
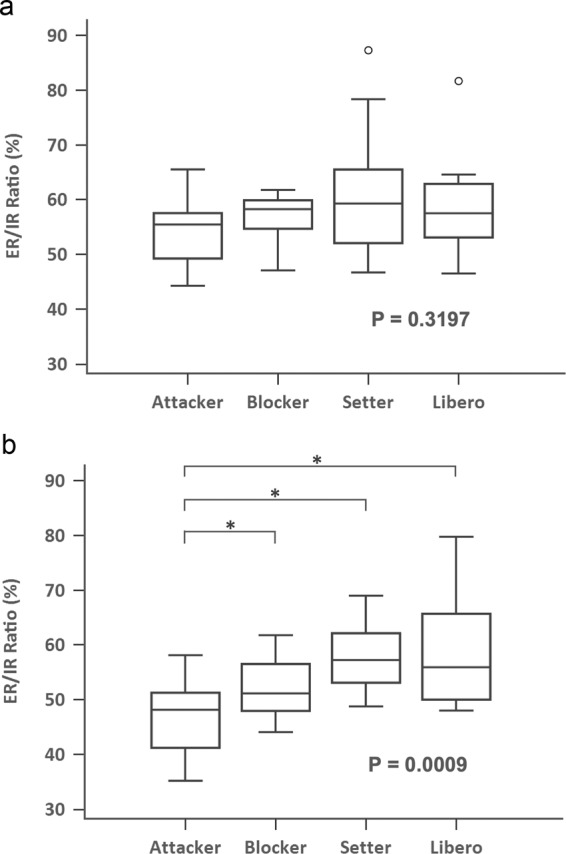
Box plot comparing ER/IR ratio for different position of volleyball players at 60 degree/s (**a**) non-dominant shoulder (P = 0.3197, Kruskal-Wallis test) (**b**) dominant shoulder (P = 0.0009, Kruskal-Wallis test; ^*^P < 0.05, post-hoc test) (ER, external rotator; IR, internal rotator).

**Figure 2:**
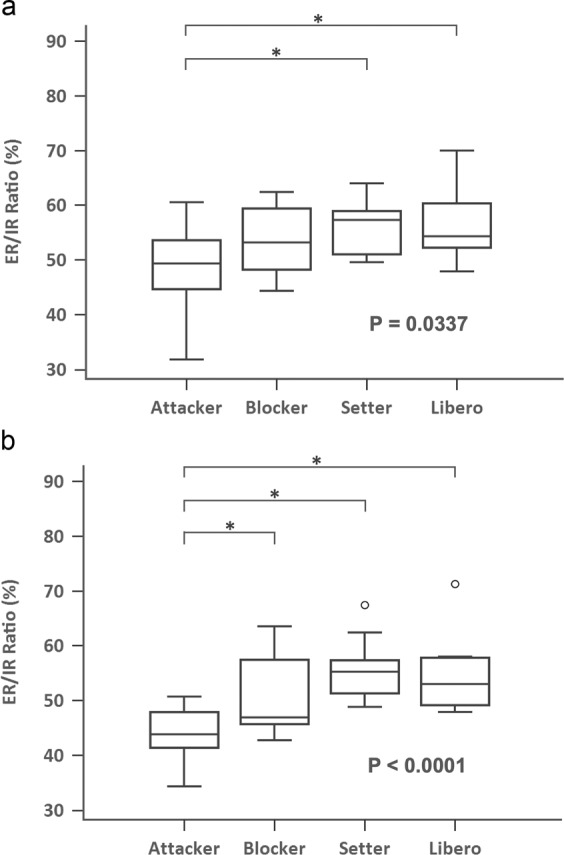
Box plot comparing ER/IR ratio for different position of volleyball players at 180 degree/s (**a**) non-dominant shoulder (P = 0.0337, Kruskal-Wallis test; *P < 0.05, post-hoc test) **(b)** dominant shoulder (P < 0.0001, Kruskal-Wallis test; *P < 0.05, post-hoc test) (ER, external rotator; IR, internal rotator).

When comparing the ER/IR ratio between different playing positions, the non-dominant side showed no ER/IR ratio difference at 60°/s (P = 0.3197, Fig. 1a), but showed significant ER/IR ratio difference at 180°/s (P = 0.0337, Fig. 2a). When evaluating the intergroup differences by post-hoc test, the attacker position had a significant smaller ER/IR ratio than setter and libero positions at 180°/s on the non-dominant shoulder (Fig. 1b). There was a significant difference of ER/IR ratio in the dominant shoulder between four different positions at both 60°/s and 180°/s (P = 0.0009, P < 0.0001, respectively, Figs. 1b, 2b). When evaluating the intergroup differences by post-hoc test, the attacker position had a significant smaller ER/IR ratio than all the other three positions at 60°/s on the dominant shoulder (Fig. 1b). Moreover, the attacker position also had a significant smaller ER/IR ratio than the setter and libero positions at 180°/sec (Fig. 2b).

## Discussion

This study evaluated the shoulder muscle strengths in male healthy volleyball players without shoulder injuries. Our results showed that the IR strength of the dominant shoulder was higher than that of the non-dominant shoulder for most of all positions of players, while the ER strength was not different between dominant and non-dominant shoulders in all positions. By the nature of volleyball, the overload on the surrounding tissue of shoulder joint from the main usage and excessive external rotation of the dominant shoulder during the spike motion caused an imbalance between the dominant and non-dominant side. As a result, such repetitive movements are known to cause shoulder instability during movement^8,13^. These muscle strength imbalances, which may cause shoulder injuries, can now be easily and accurately assessed using isokinetic muscle strength tests^7^.

The results of our study showed that the IR muscle strength of the dominant side is significantly higher than that of the non-dominant side for all positions except libero. These results were consistent with those of previous studies in which the dominant side had 3–9% greater IR strength than the non-dominant side for overhead athletes^13,14^. The side-to-side difference in shoulder IR strength may also be accused from the regular training. Most volleyball players use one arm as the dominant arm to practice forceful spikes, and overhead serves during the training season. Although ERs and IRs are both related to these two important movements in the volleyball, IR concentric strength correlates more with volleyball spike velocity^15^. Concentric training has been shown to increase concentric and eccentric strength, but eccentric training does not increase concentric strength^16^.

In particular, the peak torque of strikers’ IR strength was the highest, indicating that repetitive spike motions may increase the internal rotation strength. Also, a previous study of volleyball players reported that the ER strength of the dominant side was 0–14% weaker than that of the non-dominant side^13^. In our study, at 60°/sec, only the attacker had slightly weaker ER muscle strength of the dominant shoulder than that on the non-dominant shoulder, though not reaching a statistical difference. Moreover, although blocker did not have a significant lower ER strength, the ER/IR ratio on the dominant shoulder was still significant different from non-dominant shoulder due to the stronger IR strength. Besides, unlike the attacker position, the setter and libero positions did not show the significant differences in ER on the dominant side and had the ER/IR ratios within the normal range. Despite playing the same sport, the reason for this difference is that liberos and setters make fewer spike motions, which can cause shoulder injuries, compared with the attackers. Moreover, these positions usually require the use of both hands to pass the ball and make repetitive defense movements. The attacker and blocker positions require repeated spike motions, which mainly involve concentric internal rotation and eccentric external rotation. As a result, repetitive training of the spike motion increases the possibility of muscle damage and degeneration due to eccentric overload^8,17^. This may be the reason why the IR muscles of the dominant arm become strong, and the ER muscles become weak through this kind of specific training^2,18^.

In the previous studies, the results on the shoulder ER strength asymmetry were conflicted. Wang *et al*.^19^ find statistically important differences in ER strength in men, whereas Hidzic *et al*.^11^ did not. In this study, nearly 80% of participants had normal strength asymmetry of the ERs (ie, strength difference within 15%), as proposed by Reinold *et al*.^20^. Our result was consistent with the finding of a recent study by Hidzic *et al*.^11^ in which the side-to-side difference of ER strength was not statistically significant in 63% of male volleyball players without a history of injuries^11^. These findings may have resulted from our subject selection of volleyball players without shoulder injuries and the team’s continuous training of the IR and ER muscles and scapular stabilizers to prevent injury and provide dynamic shoulder stability.

The overall results of this study showed that the IR strength significantly improves while ER strength decreases for the attacker and blocker positions, which involve many overhead motions^5,6^. Of all muscles of the shoulder, evaluating shoulder ER and IR is most informative because those muscle groups are responsible for dynamic stabilization of the glenohumeral joint^2,21^. Furthermore, the ER/IR strength ratio was used to indicate optimal dynamic shoulder stability in overhead athletes^7^. The lower ER/IR ratio and reduced strength of the ER muscles (supraspinatus, infraspinatus, teres minor complex) in the dominant arm seem to suggest that the spiking action itself may evoke disproportionate concentric IR strength in the dominant shoulder that is not matched by ER eccentric strength. Concerning the shoulder muscle peak toque ratio, the attacker had a lower ER/IR ratio than the libero and setter positions, indicating a potentially increased risk of ER muscle injury. This suggests that training exercises for volleyball athletes, especially attackers and blockers, to maintain a favorable ER/IR strength balance and increase internal rotation flexibility may prevent or lessen the severity of repetitive overload injuries^7^.

This study encountered several limitations. First, this study is a cross-sectional study without long-term follow-up. Though the attacker had the most imbalanced shoulder muscle strength, we still could not confirm that this imbalanced shoulder condition would put the players at risk of a subsequent shoulder injury. Further study with a longer follow-up will be needed to validate this issue. Second, this study only included male volleyball players. The results of this study could not be extended to female volleyball players. We did not know whether this kind of distribution of shoulder asymmetry also existed among different positions of female volleyball players. Third, we did not perform eccentric strength testing. Though with the concerns of reproducibility in the testing^22^, the eccentric strength of the IRs for shoulder stabilization during the arm cocking phase of a volleyball spike and the eccentric strength of the ERs for the arm-deceleration phase were both important for volleyball players^10^. We might add this in future studies. In conclusion, all positions of volleyball players had asymmetric shoulder eccentric muscle strength, especially attacker, in this study. This imbalance of shoulder external rotators and internal rotator might put them at risk of shoulder injuries. The training should focus on strengthening of external rotators of the shoulder for elite volleyball players, especially for attacker and blocker.

## Methods

### Participants

We enrolled male healthy professional volleyball athletes from Samsung Bluefangs volleyball team in Korean V-league in this cross-sectional study. The participants should be first-year rookie, healthy athletes, and be medically cleared for participation by an orthopedic specialist. We excluded players with any significant shoulder injury or surgical history. Participants with shoulder conditions other than shoulder impingement syndromes, such as rotator cuff tears, adhesive capsulitis, shoulder tendinitis, and other shoulder pathologies, were also excluded.

All research procedures were reviewed and approved by the bioethical committee of the Samsung Medical Center (SMC 2019-03-120), and the study conformed to the tenets of the Declaration of Helsinki for medical research involving human subjects. All subjects received a clear explanation of the study, including the risks and benefits of participation and provided written informed consent for the testing and data analysis before the beginning of the study.

### Isokinetic testing

We evaluated the isokinetic strengths and peak torques of the IR and ER muscle strengths of the bilateral shoulders as well as the IR/ER ratios using a Cybex CSMI isokinetic dynamometer. To ensure quantitative and accurate data of muscle strength and left to right differences, all tests were measured by the same qualified person, who was familiar with a Cybex CSMI isokinetic dynamometer.

Concentric shoulder external/internal peak torques were measured at angular velocities of 60°/s and 180°/s. Participants performed three submaximal familiarization trials. Moreover, then, they performed maximal concentric IR and ER muscle strength tests. We provided standardized and consistent oral encouragement such as “push as hard as possible” and “push as fast as possible.” After a 5-min break, the test was repeated on the other shoulder using the same protocol^12,17^. The sequence of shoulders for examination was randomized. The peak torques (PT) generated from the isokinetic dynamometer were normalized to each participant’s body weight (BW), and were expressed as N-m/Kg^23^. The ER/IR ratio of the dominant and non-dominant extremities were calculated for the analysis.

### Statistical analysis

All data were analyzed using SPSS version 18.0 (SPSS Inc., Chicago, IL, USA). Means (SD) were calculated for different parameters. Wilcoxon signed-rank test was used to compare the muscle strength and ER/IR ratio between the dominant and non-dominant shoulders in the same participant. Kruskal-Wallis with the post-hoc Dunn test was used to compare the concentric ER/IR ratio of isokinetic strength between players with different positions. The level of statistical significance was set at 0.05.

## Data Availability

The datasets generated during and/or analysed during the current study are not publicly available due to the regulation of ethics institution of Sungkyunkuwan University but are available from the corresponding author on reasonable request.

## References

[1] Kilic O, Maas M, Verhagen E, Zwerver J, Gouttebarge V (2017). Incidence, aetiology and prevention of musculoskeletal injuries in volleyball: A systematic review of the literature. Eur. J. Sport. Sci..

[2] Bahr R, Reeser JC (2003). Injuries among world-class professional beach volleyball players. The Federation Internationale de Volleyball beach volleyball injury study. Am. J. Sports Med..

[3] de Ruiter CJ, de Korte A, Schreven S, de Haan A (2010). Leg dominancy in relation to fast isometric torque production and squat jump height. Eur. J. Appl. Physiol..

[4] Agel J, Palmieri-Smith RM, Dick R, Wojtys EM, Marshall SW (2007). Descriptive epidemiology of collegiate women’s volleyball injuries: National Collegiate Athletic Association Injury Surveillance System, 1988–1989 through 2003–2004. J. Athl. Train..

[5] Wagner H (2014). Upper-body kinematics in team-handball throw, tennis serve, and volleyball spike. Scand. J. Med. Sci. Sports..

[6] Lin DJ, Wong TT, Kazam JK (2018). Shoulder Injuries in the Overhead-Throwing Athlete: Epidemiology, Mechanisms of Injury, and Imaging Findings. Radiology..

[7] Stickley CD, Hetzler RK, Freemyer BG, Kimura IF (2008). Isokinetic peak torque ratios and shoulder injury history in adolescent female volleyball athletes. J. Athl. Train..

[8] Andrade Mdos S, Fleury AM, de Lira CA, Dubas JP, da Silva AC (2010). Profile of isokinetic eccentric-to-concentric strength ratios of shoulder rotator muscles in elite female team handball players. J. Sports Sci..

[9] Clarsen B (2015). The prevalence and impact of overuse injuries in five Norwegian sports: Application of a new surveillance method. Scand. J. Med. Sci. Sports..

[10] Asker M (2018). Risk factors for, and prevention of, shoulder injuries in overhead sports: a systematic review with best-evidence synthesis. Br. J. Sports Med..

[11] Hadzic V, Sattler T, Veselko M, Markovic G, Dervisevic E (2014). Strength asymmetry of the shoulders in elite volleyball players. J. Athl. Train..

[12] Silva M, Sattler T, Lacerda D, João PV (2016). Match analysis according to the performance of team rotations in Volleyball. Int. J. Perform. Anal. Sport..

[13] Witvrouw E (2000). Suprascapular neuropathy in volleyball players. Br. J. Sports Med..

[14] Wang HK, Macfarlane A, Cochrane T (2000). Isokinetic performance and shoulder mobility in elite volleyball athletes from the United Kingdom. Br. J. Sports Med..

[15] Forthomme B, Croisier JL, Ciccarone G, Crielaard JM, Cloes M (2005). Factors correlated with volleyball spike velocity. Am. J. Sports Med..

[16] Mont MA, Cohen DB, Campbell KR, Gravare K, Mathur SK (1994). Isokinetic concentric versus eccentric training of shoulder rotators with functional evaluation of performance enhancement in elite tennis players. Am. J. Sports Med..

[17] Michael J, Konig D, Hessling U, Popken F, Eysel P (2003). Results of shoulder isokinetic testing in volleyball players. Sportverletz. Sportschaden..

[18] Schons P (2019). The relationship between strength asymmetries and jumping performance in professional volleyball players. Sports Biomech..

[19] Wang HK, Cochrane T (2001). Mobility impairment, muscle imbalance, muscle weakness, scapular asymmetry and shoulder injury in elite volleyball athletes. J. Sports Med. Phys. Fitness..

[20] Reinold MM, Gill TJ (2010). Current concepts in the evaluation and treatment of the shoulder in overhead-throwing athletes, part 1: physical characteristics and clinical examination. Sports Health..

[21] Lee SB, Kim KJ, O’Driscoll SW, Morrey BF, An KN (2000). Dynamic Glenohumeral Stability Provided by the Rotator Cuff Muscles in the Mid-Range and End-Range of Motion: A Study in Cadavera. J. Bone Jt. Surg. Am..

[22] Malerba JL, Adam ML, Harris BA, Krebs DE (1993). Reliability of dynamic and isometric testing of shoulder external and internal rotators. J. Orthop. Sports Phys. Ther..

[23] Jaric S, Mirkov D, Markovic G (2005). Normalizing physical performance tests for body size: a proposal for standardization. J. Strength. Cond. Res..

